# Editorial Notes and Miscellany

**Published:** 1911-12

**Authors:** 


### Editorial Notes and Miscellany.

Dr. W. T. Jones, a long time practicer of medicine at Georgetown, Texas, fell dead in his office November 13th ult.

---

Six Hundred young women are students in the University of Texas. Eleven are studying medicine and one is studying law.

---

At the Stated monthly meetings of the New York Polyclinic Medical Society the proceedings are carefully taken by stenographer, condensed, and typewritten.

---

The Abstract of the article upon "Pneumonitis," which occurred in the November issue of this journal, should have been credited in the September issue of the *Medical Brief*.

THE TRULY GREAT WALTER WYMAN is dead—the able and most efficient Surgeon General of the Public Health and Marine Hospital Service. This is a great loss to the nation—a calamity.

---

CLUB.—TEXAS MEDICAL JOURNAL, one year, $1.00; *American Journal Clinical Medicine,* $2.00; will send both journals for $2.00; will add Dr. Daniel's book, "Dr. Bruno," for $1.00 more. Here you get $3.00 for $2.00, or $4.50 for $3.00.

---

THE INCREASE for this year of the class beginning to study medicine in the University of Texas at Galveston is nearly 50 per cent. This increase has been made despite the fact that no student is now admitted to study medicine who has not done a full year of satisfactory work in a first-class college.

---

DR. R. P. TALLEY of Temple, Texas, died at his home October 30, 1911, after a long illness, aged 75. Dr. Talley was a leading physician in Bell county forty odd years. He was formerly an active member of the State Medical Association, but in later years he dropped out. He was a lieutenant in the Confederate army.

---

ARMY MEDICAL CORPS EXAMINATIONS.—The Surgeon General of the Army announces that preliminary examinations for the appointment of first lieutenants in the Army Medical Corps will be held on January 15, 1912, at points to be hereafter designated. Full information concerning these examinations can be procured upon application to the "Surgeon General, U. S. Army, Washington, D. C."

---

DR. R. H. L. BIBB, a noted physician and surgeon, has returned to his first love, Texas, and opened an office at Austin. Some thirty years ago he was a resident of Austin and secretary of the State Medical Association. He took up his residence in Saltillo, Mexico, where he has continuously resided now twenty-nine years. In Mexico he was at one time chief surgeon of all the railroads in the republic, but had to resign the office on account of his health. His hosts of friends in Texas will welcome him back.

---

DR. MALONE DUGGAN, of San Antonio, Texas, the President of the State Association of Social Hygiene, delivered a lecture at Carnegie Hall in San Antonio, Saturday evening, November 25th

(ult.), before a brilliant audience. His lecture was entitled "Fruition," and was beautifully illustrated by stereopticon slides, showing the effects of heredity in the offspring of marriage of the unfit, and numerous other factors in the decay of the human race, alcohol, venereal disease, child labor, etc. Dr. Duggan is doing splendid missionary work *con amore,* and he will lecture anywhere invited. His example should be followed by physicians in every county in Texas, the lecturers to be selected in rotation by the president of each county medical society. Only in that way can the people be educated in eugenics and awakened to the necessity of marriage laws and *sterilization* of the insane and hopeless criminals.

---

Dr. C. O. Weller, a long time (twenty-seven years) practicer of medicine in Austin, died of pneumonia at his home, November 10th ult., aged 70. He was an ex-Confederate soldier, Fifth Texas Cavalry, Green's Brigade; born in Tipton county, Tennessee, November 12, 1842. Settled at Victoria, Texas, in 1855, from which place he entered the Confederate army. After the war he studied medicine one year at the University of Louisiana, now Tulane, and then at Jefferson Medical College, graduating an M. D. in 1869. He then located at Columbus, Texas, and moved to Austin in 1884. His wife was Florence Buford. Mrs. Weller survived her husband only two days. She died of pneumonia also. The parents are survived by two daughters and three sons,—one, Dr. C. Burford Weller, who succeeds his father in practice at Austin; Clarence W., now studying medicine at the State University Medical College, and McLeary Weller, a well known newspaper man in New York. Dr. Weller was greatly esteemed by all who knew him.

---

Semi-Annual Meeting of Fifth District (Bexar) Medical Association.—A most interesting meeting of this famous society was held in San Antonio, Thursday, November 24th (ult.), at which the seventeen counties composing it were represented. Dr. J. S. Lankford, of San Antonio, delivered an extemporaneous address on "Sanitation on the Panama Canal," which was listened to with close attention, and loudly applauded. Dr. W. B. Russ, of San Antonio, late President State Medical Association, had a fine paper on "Surgery of the Colon"; Dr. B. F. Stout, San Antonio, demostrated rebreathing of nitrous oxide as a general anesthetic. Dr. F. D. Boyd, of Fort Worth, read a paper on "Salvarsan in Relation to Diseases of the Eye." Dr. C. S. Venable, a paper on "Hyperthyrodism from a Surgical Standpoint," which elicited much interest and brought out much learning, some of which *we* thought "ain't so,"—one gentleman speaking of the *duct* of the thyroid and the control of the sexual function in the male by the secretion of this gland.

Officers elected: Dr. W. T. Dawes, Gonzales, President; Dr. H. H. Ogilvie, San Antonio, Secretary, and Dr. W. C. Williams, of Staples, Treasurer, succeeding Dr. E. V. De Pew, President; Dr. F. C. Walsh, Secretary, and Dr. Wile, of New Braunfels, Treasurer.

---

RICHLAND SPRINGS, TEXAS, October 19, 1911.

*Dr. F. E. Daniel, Austin, Texas:*

Here goes for another year. Keep up the good work. May you be able to wield a fearless pen many years longer; and I am sure you will always be found on the side against "one man rule."

Yours fraternally,

A. D. NELSON.

---

WINNSBORO, TEXAS, October 28, 1911.

*Dr. Daniel.*

MY DEAR FRIEND: Please find $1.00, pass to our credit. Wishing you much success and may you continue to be useful in the future as you have been in the past, is the sincere wish of among the oldest subscribers to the "Red Back."

T. N. SKEEN.

(Twenty-seventh year.)

---

TURTLE BAYOU, TEXAS, October 19, 1911.

*Dr. F. E. Daniel, Austin, Texas.*

DEAR DR. DANIEL: I am sending check for $5.00 to pay up subscription to the "Red Back," which I esteem highly as one of our home medical journals. I have been a subscriber to your journal for eighteen years, and am convinced that Osler made a mistake when he said, "Man's usefulness ended at the age of 45." You may send me by mail your book, "The Strange Case of Dr. Bruno." Wishing you many more years of successful journalism, and with best regards to Bettie and the baby, I am,

Very truly,

GEO. L. MORGAN.

